# Canavanine Content Quantification in Processed Bitter Vetch (*Vicia ervilia*) and Its Application as Flour in Breads: An Analysis of Nutritional and Sensory Attributes

**DOI:** 10.3390/foods13162528

**Published:** 2024-08-14

**Authors:** Adi Nudel, Shahal Abbo, Zohar Kerem

**Affiliations:** The Levi Eshkol School of Agriculture, The Hebrew University of Jerusalem, Rehovot 7610001, Israel; adi.nudel@mail.huji.ac.il (A.N.); shahal.abbo@mail.huji.ac.il (S.A.)

**Keywords:** grain legume, nutritional value, resilient-crop, PDCAAS

## Abstract

Bitter vetch (*Vicia ervilia* Willd.) is a traditional Mediterranean–West Asian legume, mainly used as livestock feed because of its toxic non-proteinogenic amino acid, canavanine. However, historical sources suggest its past human consumption. Currently, bitter vetch is a minor crop confined to marginal soils in semi-arid regions, presenting a potential alternative protein source amid projected climate changes. This study evaluated the nutritional and sensory attributes of bitter vetch seeds processed through various household methods. Germination and cooking significantly reduced the canavanine content by 28% and 60%, respectively. Incorporating bitter vetch flour (BVF) into wheat bread enhanced protein and fiber contents without substantially altering carbohydrate and lipid levels, and the baking process reduced the canavanine content by 40%. Bitter vetch flour enriched the bread with iron and calcium, contributing significantly to their daily nutritional intakes. Sensory evaluations indicated positive reception for bread with 12% BVF, achieving a balance between nutritional enhancement and consumer acceptance. This study identifies bitter vetch seeds as a valuable resource for improving bread formulations with corrected gluten contents and enhanced protein quality, as measured using protein-digestibility-corrected amino acid score (PDCAAS) values. With strategic processing and formulation adjustments, bitter vetch has the potential to re-emerge as a feasible high-protein grain crop, promoting sustainable farming.

## 1. Introduction

Bitter vetch (*Vicia ervilia* (L.) Willd.), a member of the Papilionaceae (Fabaceae) family, is a traditional minor crop across the Mediterranean Basin and West Asia. Unlike the other Near-Eastern founder crops, bitter vetch is mostly a feed crop, which grains (and straw) are mostly fed to livestock because they contain canavanine, a toxic non-proteinogenic amino acid [1]. Canavanine (L-2-amino-4-guanidooxy-butanoic acid) is one of over 1000 nonproteinogenic amino acids synthesized in plants [2]. Many species produce this natural product abundantly as a potent chemical defense against predation and disease. Canavanine, a natural homolog of l-arginine, can replace it during protein synthesis, resulting in aberrant canavanyl proteins. Indeed, its antitumor activity was demonstrated both in vitro and in vivo cancer research [3,4,5,6].

Canavanine occurs naturally in various human food sources, including beans, onions, and alfalfa seeds and sprouts, with concentrations ranging from 2.1 ppm in soy flour to as high as 10,000 ppm in onions [7]. Soaking in water, leaching, and boiling were shown to render bitter vetch grains palatable [8]. Previous studies examined the influences of different soaking methods on antinutrient contents, showing that aqueous and saline treatments were favorable for canavanine reduction [9] and that the effect of extrusion cooking was useful in reducing the canavanine content while improving protein and starch digestibilities [10]. To that end, it is important to determine the initial canavanine contents in a wide array of domesticated cultivars and to assess the influences of different processing and cooking methods on its contents.

Ethnographic sources confirm the human consumption of bitter vetch (BV) during food shortages [11]. Recent stable isotope analyses have shown its importance as a staple crop in the Iron Age in western–central Italy, though it is absent from modern European diets [12]. Traditional (past subsistence) agriculture prioritized yield stability and crop diversity to meet local food and feed needs. However, agricultural modernization led to the abandonment of many traditional crops in favor of high-yielding cash crops [13,14,15]. With global warming affecting staple crop yields, developing climate-resilient crops through the wider use of underutilized species, like bitter vetch, is suggested as a strategy to address food security challenges [16,17,18]. Broadening BV cultivation may be a promising solution when considering sustainable economic development and the need for the diversification of modern farming systems [19]. It also demonstrates new possibilities for including its grains as a modern food ingredient.

As a globally consumed staple food, bread serves as an essential source of nourishment, particularly for those in developing nations and low-income population sectors in developed countries. Given the potential of bitter vetch seeds as a high-protein flour, investigating their impacts on breadmaking processes may yield valuable insights into enhancing the nutritional profiles of common foods by the addition of this fundamental dietary component. Previous research on other regularly consumed legumes showed a 50% reduction in canavanine levels in sword beans following overnight soaking and boiling [20] and an 85–95% reduction in jack beans by roasting at medium or high temperatures [21]. The inclusion of bitter vetch flour (hence, BVF) as a minor dough component in cereal bread to enhance its nutritional quality may overcome undesirable symptoms [22,23]. To the best of our knowledge, there has been no report to date on the effects of various household processing methods, including soaking, pressure cooking, and sprouting, on the canavanine content in bitter vetch.

Nowadays, consumers are aware of the benefits of a healthy diet, in terms of caloric consumption, protein, and dietary fiber, when facing two opposite, yet equally distressing, phenomena: obesity and malnutrition. One of the strategies suggested to increase nutrition security is the utilization of climate-resilient crops to produce attractive and nutritious bread products [24]. Numerous works have evaluated the effects of different legume flour enrichments on bread’s characteristics [25,26], but, to the best of our knowledge, this is the first modern report on the incorporation of BVF into breads.

Methods for the analysis of canavanine in plant samples have evolved over the years. In the past, spectrophotometric methods were used [27], and, nowadays, most methods are based on the precolumn derivatization of amino acids with different reagents, such as ortho-phthalaldehyde (OPA) [28], dansyl chloride [29], and diethyl ethoxymethylenemalonate (DEEMM) [30], as was performed in this work.

The goals of this research were as follows: (a) to determine the effects of various household processing methods on the canavanine content of bitter vetch seeds and (b) to evaluate the effects of BVF addition on the nutritional value and the acceptability of wheat bread.

## 2. Methods

### 2.1. Chemicals

All the solvents and chemicals were of HPLC or analytical grade. Water was passed through a Milli-Q water purification system (Millipore, Bedford, MA, USA). Acetonitrile, canavanine, and DL-2-aminobutyric acid (internal standard, I.S.) were obtained from Sigma–Aldrich (St. Louis, MO, USA). Diethyl ethoxymethylenemalonate was obtained from Acros Organics (Geel, Belgium). 

### 2.2. Seed Samples

Bitter vetch is a relic crop in Israel. Local landraces (no modern cultivars) are grown on marginal land by traditional farmers in Judea and Samaria. Seeds were sourced from a traditional farming community in Samaria via mediation by Eden Seeds Co. (https://1145-il.all.biz/, accessed on 8 August 2024) from the summer 2021 harvest. The seeds were cleaned and kept in vacuumed bags at room temperature until processing. A number of homozygous germplasm lines are held in our laboratories for the sake of future investigations and serve for the seed canavanine contents’ determination.

### 2.3. Processing

Different processing methods included (1) finely ground seeds, (2) seeds soaked in water (1:5, wt./vol.) at room temperature for 12 h, (3) seeds soaked and then germinated at room temperature for 24 h, (4) seeds cooked in boiling water, (5) seeds pressure cooked at 120 °C, and (6) bread amended with BVF and baked at 180 °C. The seeds were finely ground using an IKA A11 analytical mill for 60 s (IKA, Staufen, Germany) for treatments (1) and (6) and prior to the analysis for all the other treatments. All the samples were dried for 48 h at 60 °C prior to the analysis.

### 2.4. Basic Analysis

The samples (in 3 replicates for each treatment) were dried in a forced-air oven at 60 °C for 48 h and ground in an IKA mill to pass through a 1 mm screen. The AOAC standard procedures were used to determine dry matter [31].

### 2.5. Bread Preparation

Bread was prepared according to the method described previously [32]. The dough was kneaded in a 5 quart (4.75 L) KitchenAid mixer (KitchenAid Portable Appliance, St. Joseph, MI, USA), proofed at 50 °C for 1 h in an oven (Blue M Electric Company, Blue Island, IL, USA), shaped and proofed again for 20 min, and then baked at 180 °C for 45 min. All the recipes contained wheat flour (543 g), olive oil (21 g), sugar (40 g), salt (10 g), dry yeast (9 g), and water (377 g). The weight of the dough after shaping was 510 g. Four formulations were prepared, containing 0, 12, 25, or 40 g of raw bitter vetch flour per 100 g of total flour. Gluten was added to the bitter-vetch-enriched breads at the needed amount, completing the gluten reduction by replacing wheat flour with BVF. The wheat flour contained 1.6% ash and 12% gluten.

### 2.6. Determination of Nutritional Composition

For the macronutrient analysis, the total protein (AOAC 978.04), fat (AOAC 920.85), and ash (AOAC 923.03) were determined using standard analytical methods, as described by American Organization of Analytical Chemists International (AOAC International) procedures [33]. The dietary fiber content was determined using the enzymatic–chemical method, in accordance with AOAC 994.13 [33]. The total carbohydrates were calculated by subtracting the moisture, protein, fat, and ash from the total weight of the food, and the energetic contribution was calculated using Equation (1) as follows:

(1) $$Energy\;\left(Kcal\right)=4\times \left(protein\;\left(g\right)+carbohydrate\;\left(g\right)\right)+9\times \left(fat\;\left(g\right)\right)$$

Equation (1): Calculation of energy in *Kcal*.

### 2.7. Calculations of Protein-Digestibility-Corrected Amino Acid Scores (PDCAASs)

The WHO/FAO/UNU essential amino acid scoring pattern for 1- to 2-year-old children [34] was used as a guideline. PDCAAS values, expressed as percentage units, were calculated as follows: comparing the published profiles of the amino acids (AAs) of the BV and all the other ingredients to that of a reference standard, assessing the limiting AA’s lowest rate (AA in the food product/AA in the reference standard), and multiplying the score of the limiting amino acid with the in vitro protein digestibility. This calculation was performed for each ingredient, according to the literature (Table 1), and used to determine each bread’s score. The protein digestibility of the BVF was estimated to be 0.5 because it is a plant protein, although it is recognized that processing may enhance its digestibility [35].

### 2.8. Determination of Mineral Composition

A 2 g sample was subjected to acid digestion using an Ethos Easy microwave laboratory oven (Milestone, Sorisole, Italy), in Teflon vessels with HNO_3_ and H_2_O_2_ (30%, Merck, Darmstadt, Germany). Yttrium (Y) was added to all the samples and controls as an internal standard. After digestion, the samples were dissolved completely with deionized water (Milli-Q, <18 MΩ·cm, Millipore, MA, USA). The clear solutions were analyzed using an HR Dual-View ICP-OES spectrometer instrument (PlasmaQuant 9000 Elite, Analytik Jena, Jena, Germany) to determine the concentrations of the different elements [39]. The test was performed in triplicate, and the results were expressed as milligrams per kilogram of bread.

### 2.9. Sensory Evaluation of the Bitter Vetch Breads

Twenty untrained panelists, 14 females and 6 males, aged from 25 to 60 years, who regularly consume wheat bread, were recruited for the descriptive sensory evaluation of the bitter vetch breads. The panelists were familiarized with the sensory descriptors for bread, verbal definitions of the attributes, and the evaluation scales. The sensory evaluation was carried out by evaluating 7 major sensory attributes, such as appearance, taste, bitterness, aroma, and overall acceptability, using a 9-point hedonic scale [40]. Four bread formulations were tasted by each panelist, and all the testers involved in the sensory analysis were informed about the aims of the study. Each panelist was given one full slice (of a 400 g loaf) presented on a tray. Lukewarm water was served as a palate cleanser between the samples. This research was approved by the Hebrew University ethics committee. 

### 2.10. Extraction of Canavanine

Free canavanine was extracted as was previously described [30] with minor modifications. Samples (10.0 mg in 1.5 mL reaction tubes) were sonicated in ethanol:water (3:7 *v*/*v*, 200 µL) for 15 min at room temperature using an Elma sonicator bath (Singen, Germany) and centrifuged at 10,000× *g* for 10 min using a Sigma microcentrifuge (Osterode am Harz, Germany). Pellets were re-extracted once more, and the resulting supernatants were pooled. 

### 2.11. Precolumn Derivatization

For the detection of the amino acids under UV derivatization, diethyl ethoxymethylenemalonate (DEEMM) was used. The internal standard (5 mM, 10 µL) and DEEMM (2 µL) were added to a tube containing 900 µL of 1 M borate buffer (pH 9.0) and 100 µL of the sample. The solution was mixed thoroughly and incubated at 50 °C for 50 min. Samples were filtered through 0.45 µm PTFE membranes before injection into the HPLC system (30 µL).

### 2.12. HPLC Analysis

For the analysis of the derived canavanine, a reverse-phase Luna C-18(2) 5 μm × 3.9 × 300 mm column (Phenomenex, Milford, MA, USA); a JASCO HPLC (LC-2000, Nagoya, Japan) system consisting of a columnar oven (JASCO, CO-2060, Tokyo, Japan), a UV–Vis diode array detector (JASCO, MD-2010DAD, Tokyo, Japan) set at 220 nm, and a liquid chromatography pump (JASCO, PU-980, Tokyo, Japan); and a ChromNAV 2.0 software program (JASCO, Tokyo, Japan) were used. The column’s temperature was 30 °C.

### 2.13. Statistical Analyses

The data were analyzed using one-way ANOVA, and the mean values were compared using Student’s *t*-test. All the statistical analyses were performed using JMP 15 software.

## 3. Results and Discussion

### 3.1. Bitter Vetch’s Nutritional Value

The nutritional profile of the raw BV seeds was analyzed and compared to published data [41] of three major food legumes, namely, chickpeas, green lentils, and dried peas (Table 2). The results demonstrate the resemblance between the nutritional contributions of the traditional food legumes of the Mediterranean Basin, mainly green lentils and BV. Green lentil flour has emerged as an innovative ingredient within the food technology industry, garnering interest from food technologists and consumers alike [42]. This flour has gained popularity because of its exceptional and well-balanced nutritional profile, as well as its ability to enhance the taste and texture of various food products [25,43]. In this study, we demonstrate that bitter vetch flour exhibits a comparable nutritional value to that of the green lentil flour currently used by the food industry. Hence, we reiterate the potential of bitter vetch as an alternative ingredient and raw material, particularly because of its stable yields in marginal farming systems under global warming and changing climate patterns.

### 3.2. Effects of Processing on Canavanine Content

Traditionally, canavanine has been considered as an antinutritional compound because it tends to substitute arginine in de novo-synthesized proteins. Recent investigations have unveiled a multifaceted repertoire of bioactive properties associated with canavanine. Of particular interest are its antiproliferative, chemopreventative, chemosensitizing, and radiosensitizing effects [44]. This paradigm shift has redefined canavanine as a bioactive compound with the capacity to promote health and offer therapeutic benefits [5,45]. It is produced by many legumes, including jack bean and lucerne (alfalfa), and is accumulated mainly in seeds and present in germinating sprouts [30]. Still, it is generally accepted that bitter vetch is not a human food because of its seeds’ canavanine content [46]. In retrospect, the actual canavanine content following processing, as well as those in other acceptable human foods (e.g., alfalfa sprouts for salads), may suggest that the status of the bitter vetch and its use in human foods need to be reconsidered.

**Figure 1:**
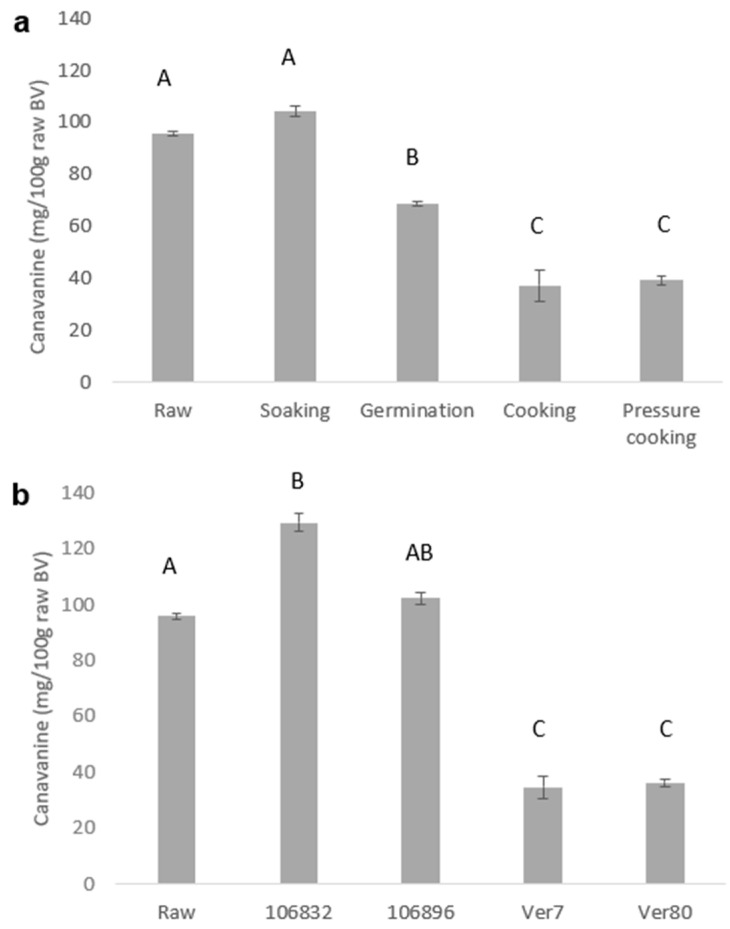
(**a**) Canavanine contents (mg/100 g of raw bitter vetch) in raw and processed bitter vetch seeds. Values are expressed as means ± standard deviations of triplicate determination. Means with the same letters are not significantly different (*p* < 0.05). (**b**) Canavanine contents (mg/100 g of raw bitter vetch) in several homozygous bitter vetch germplasm lines (106832, 106896, Ver7, and Ver80) relative to that in the traditional raw seed mixture (obtained from subsistence farmers) used for all the baking and sensory experiments. Means with the same letters are not significantly different (*p* < 0.05).

The European Food Safety Authority (EFSA) issued a report regarding the safety of canavanine-containing foods that use lentils as examples of staple foods that contain 280 mg of canavanine/100 g [7], more than the concentrations in raw BV and processed BV, which are presented in Figure 1a. This safety limit makes it clear that the Israeli landrace of the BV used in this work is safe. Figure 1b presents the variability in the canavanine contents between various BV genotypes that were collected from several locations around the Mediterranean.

Bitter vetch seeds were subjected to household processing methods that are popular when preparing legumes in the kitchen. The soaking of the seeds did not reduce the canavanine content, and it remained as high as that in the raw seeds (Figure 1a). The germination of the seeds reduced the canavanine content by 28%, to a value of 68.6 mg/100 g. Heating the seeds, by either cooking or pressure cooking, reduced the seeds’ canavanine content by 60%, reaching an average value of 38.2 mg/100 g. This reduction is critical not only for the question of product safety but also for its acceptability in a market increasingly vigilant about food ingredients. Legume seeds are rarely eaten in their raw state, suggesting that canavanine levels in food would most likely be lower than that in the raw seeds.

### 3.3. Bitter Vetch Breads

**Figure 2:**
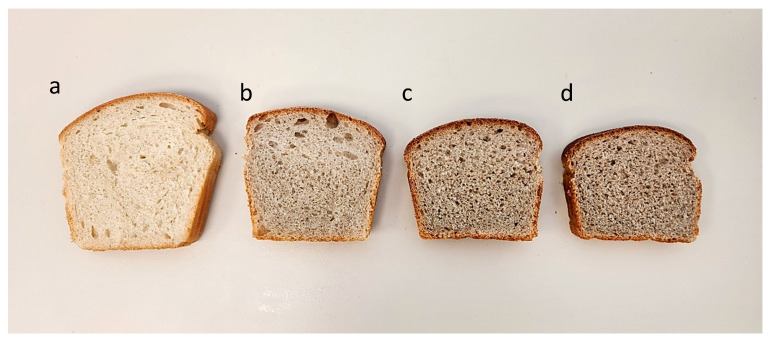
Slice appearances of (**a**) wheat bread, (**b**) 12% BVF bread, (**c**) 25% BVF bread, and (**d**) 40% BVF bread.

**Figure 3:**
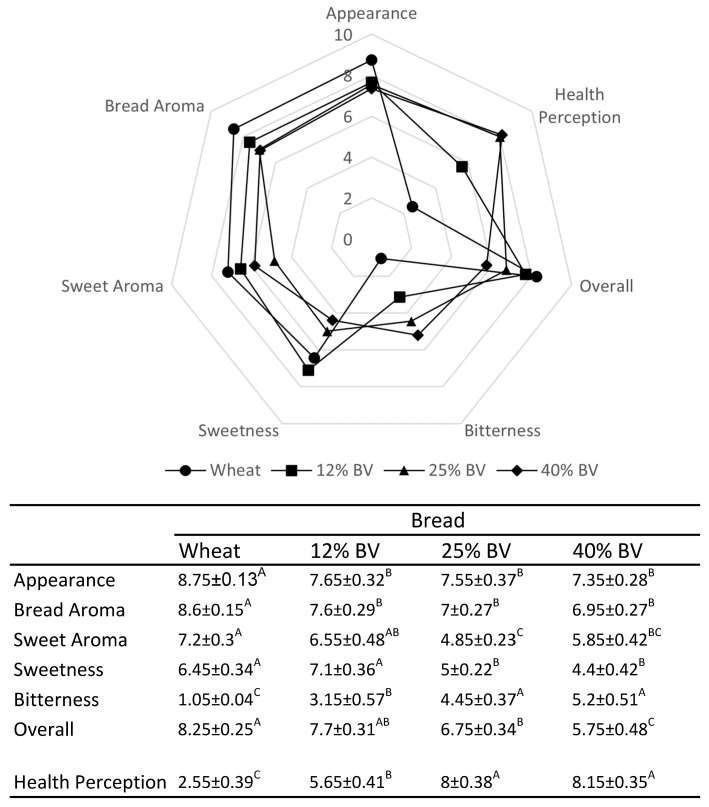
Sensory profiling of different attributes of breads supplemented with BVF. Each point represents the average of 20 panelists’ scores. Numerical data are also presented in a table, where different letters within rows indicate significant differences between bread types (*p* < 0.05; Student’s *t*-test).

Both the crust and crumb were affected by the addition of the BVF. An objective color description is usually obtained using the CIE LAB color space. In this space, the color is expressed utilizing three coordinates: L*, a*, and b*. When testing the crust, the value of parameter L*, describing the brightness of the sample, was decreased, as shown in other cases of wheat-bread amendment [47], while a* was increased, which means that the crust tended toward redness. In previous works, the dark brown color was shown to be associated with perceived overall healthiness by consumers [48], similar to our results. The crumb color darkened with increasing levels of BVF substitution (Figure 2). This probably resulted from the original darker color of the legume. The addition of the BVF had a negative effect on the loaf’s volume, which may be because of the different seed protein profile of the bitter vetch. Despite these changes in the color and density, the overall grade of the 12% BVF bread did not differ from that of the wheat bread while scoring higher in the panel’s health perception, probably because of its color (Figure 3).

When designing the formulations, we addressed the physical properties that could be negatively affected by the substitution of the gluten in wheat flour. To mitigate this, we maintained an equal amount of gluten in all four recipes. The addition of the gluten contributed to the breads’ protein contents (Table 2). However, the losses of the porosity and loaf volume were still evident (Figure 2) as the BVF percentage in the bread was elevated, indicating a denser crumb structure. It is generally known that the addition of mixed legumes to bread formulae drastically affects the loaf quality, causing a significant decrease in the bread’s volume and an increase in the air cells’ density [49]. This remains a technological challenge to be addressed.

### 3.4. Nutritional Values, Canavanine Contents, and Mineral Contents of Bitter Vetch Breads

The incorporation of the BVF and gluten into the wheat flour for the bread formulations enhances the nutritional profile in several aspects (Table 3). Adding BVF at levels of 12%, 25%, and 40%, together with the added gluten, led to increased protein contents from 6.7 to 8.3, 8.9, and 11.8 g per 100 g, respectively.

To evaluate the contributions of the new breads to the protein needs, we have calculated PDCAAS values. PDCAAS is a method used to evaluate the quality of a protein source based on two factors: its amino acid profile and how well it matches human amino acid requirements and how digestible the protein is by humans. For the calculations presented herein, we have conservatively estimated a low digestibility value of 0.5 for the bitter vetch (BV) proteins, in accordance with the literature [35]. Bitter vetch, like all other legumes, contains antinutritional factors that contribute to its low digestibility value. Still, based on BV’s high amino acid score (AAS), BVF, like most legume seed flours, qualifies to be labeled as a “good source of protein”. In this study, the added gluten has contributed more than half of the recipe’s protein content, and because of its low AAS, it makes a minor contribution to the PDCAAS value. In the design of the bread formulations in this study, we aimed to reach PDCAASs higher than those of common wheat-flour baked foods (25%). Indeed, the PDCAASs of the BVF-enriched breads were 15.8–43.5% higher compared to that of the reference wheat bread (Table 4).

The total fiber was shown to be a major component in the health promotion associated with adding the BVF to the breads. Its content has been increased from 3.8 to 5.4, 6.3, and more than doubled in the higher-BVF bread, reaching 8.3 g per 100 g of bread. The value found for the wheat bread is within the common range of common grain breads, 2–4 g of fiber per 100 g. Adding the BVF did not modify the contents of the carbohydrates and lipids, and as a result, only a slight rise in the energy content (kCal/100 g) was measured. The latter suggests a shift from available wheat carbohydrates to non-available legume fiber. Foods providing 5 g or more of fiber per serving are considered as being “high fiber”. Foods providing 8 g or more of fiber per serving are considered as excellent sources of fiber according to FDA labeling guidelines. Considering an average daily intake of 137 g per day (three–four slices [51]), the new highest-BVF bread provides 11.2 g of dietary fiber and may be labeled as an “excellent source of fiber”, providing over one-third of the recommended daily fiber (38% of the 30 g DV). Even the lowest-BVF bread baked in this study provides 7.2 g, allowing for a “high fiber” label.

The canavanine content was determined in the flour mix prior to and in the crust and crumb after baking. In all the cases, the canavanine content decreased by more than 40% (Table 4) through baking. The crust contained less canavanine than the crumb, suggesting that heat is not the sole cause of the degradation. For example, it is well established that the Maillard reaction is a profound process that differentiates the crust from the crumb [52]. The overall level of canavanine in the bread is 12 mg/100 gr, which establishes the safe daily consumption of 50–100 g of bread, i.e., two–three slices per serving [51].

Finally, the contents of all the measured minerals were also elevated in accordance with the amount of the BVF added and the initial content of the wheat flour. The calcium content was doubled and reached a level of 251 mg/Kg in the 40% BV bread. The iron content increased fourfold in the highly acceptable 25% BVF bread, up to the level of 20 mg/Kg of bread. Considering, again, an average daily intake of 137 g per day (three–four slices; U.S. Food and Drug Administration, 2018), the increased iron levels in the BV-amended breads may provide 25% and up to 40% of the 8 mg/person/day recommended allowance (RDA) for adult men and for all adults 51 years and older.

The augmentation of the wheat bread with the BVF demonstrates a promising avenue for diversifying grain-based products. The nutritional enrichment of breads through such augmentation, particularly concerning protein and mineral contents, aligns with global efforts to enhance food security through nutrient-dense foods [24]. Likewise, the considerable increase in the dietary fiber with the inclusion of the BVF offers a potential answer to growing health concerns around refined grain consumption. 

### 3.5. Sensory Evaluation of Bitter Vetch Breads

Bread loaves baked with increasing concentrations of BVF in the wheat flour were evaluated for their sensorial parameters. The addition of the BVF to the bread resulted in a slight decrease in the tasters’ evaluations of the breads’ aromas and appearances. The health perception increased considerably when evaluating the 12% BVF formulation and increased again when evaluating both the 25 and 40% BVF formulations. Evaluating the modification of the flavors shows that adding the BVF to the breads led to an increase in the bitterness, yet not to a level that disturbed the panel members. The individuals’ overall assessment of the 12% BVF bread was similar to that of the wheat bread. The 40% BVF formulation was the least favorable.

In many studies involving bitter vetch, there is a consistent reference to the high contents of alkaloids and tannins in the seeds, resulting in a bitter and astringent taste [53]. The sensory evaluation of the BVF breads showed a nuanced response to the inclusion of the BV in the breads. Although the introduction of the BVF understandably altered the sensory profiles of the breads, slightly increasing the bitterness, it did not significantly detract from the overall acceptability at lower inclusion rates. The 12% bitter vetch formulation, in particular, maintained a comparable overall preference to that of the standard wheat bread, suggesting a threshold for inclusion that maximizes the nutritional benefit, consumer acceptance, health perception, and, no less important, a safe canavanine level (Table 5).

## 4. Conclusions

The growing demand for plant proteins as sources of food and animal feed in developing countries, coupled with the rising interest in diverse substitutes for animal protein, is fueling the exploration of new sources of plant proteins. Proximal composition analysis confirmed that bitter vetch seeds are good sources of protein, fiber, and minerals. When integrating our findings with broader nutritional, agricultural, and sensory aspects, considering bitter vetch as a crop that may contribute to modern diets and sustainable agriculture seems almost inevitable.

In light of the above, we propose a re-evaluation of bitter vetch not merely as a relic of agricultural history and/or as a Near-Eastern legume domestication test case [13,15] but rather as a feasible sustainable crop with significant potential for both nutritional enrichment and agronomic resilience. The processing methods that were tested, including cooking and baking, promoted a reduction in the canavanine, demonstrating the potential to include BV in foods, whether homemade or industrially produced. Future research should focus on optimizing processing methods to incorporate BVF into other baked goods and food products, exploring the genetic variability among bitter vetch cultivars for nutritional traits, incorporating BVF into other baked goods, and expanding sensory evaluations to ensure consumer acceptance.

## Figures and Tables

**Table 1:** Amino acid scores (AASs), protein digestibilities, and calculated PDCAASs of protein sources in wheat and BVF breads [36,37,38].

Source of Protein	Limiting AA	Amount (mg/g of Protein)	FAO/WHO Ref. (mg/g of Protein)	AAS	Protein Digestibility	PDCAAS	Reference
BVF	-	-	-	1	0.5	0.50	[36]
Gluten	Lysine	83.2	362.5	0.23	0.983	0.23	[37]
Yeast	Leucine	60	66	0.91	0.68	0.62	[38]

**Table 2:** Nutritional compositions of common raw legume seeds. Bitter vetch seeds were analyzed herein, while the other values were adopted from the U.S. Department of Agriculture’s database [41].

	Legume			
Nutritional Value (g/100 g)	Bitter Vetch	Green Lentils	Chickpeas	Dried Peas
Calories/100 g	356 ± 4	356	378	364
Carbohydrates	63.6 ± 1.2	61.1	63	61.6
Lipids	1.1 ± 0.1	1.6	6.1	3. 9
Moisture	9.3 ± 0.6	9.8	7.7	8.7
Protein	22.9 ± 0.9	24.3	21.3	23.1
Ash	3.1 ± 0.1	3.3	2.9	2. 7

**Table 3:** Proximate analysis and mineral contents of BV breads. Values are expressed as means ± standard errors of triplicate determination. Within rows, means with the same letters are not significantly different (*p* > 0.05).

	Percentage BV in Flour (%)			
Nutritional Value (g/100 g)	0	12	25	40
Energy (kCal)	243.33 ± 0.87 ^A^	251.33 ± 0.32 ^B^	258.33 ± 0.87 ^BC^	255 ± 1.52 ^C^
Carbohydrates	48.76 ± 0.08	49.86 ± 0.06	50.06 ± 0.11	49.5 ± 0.15
Total Fiber	3.83 ± 0.17 ^A^	5.43 ± 0.08 ^B^	6.36 ± 0.17 ^C^	8.36 ± 0.14 ^D^
Lipids	3.25 ± 0.01	3.33 ± 0.03	3.03 ± 0.03	3 ± 0.15
Moisture	39.94 ± 0.09 ^A^	37.01 ± 0.05 ^B^	34.3 ± 0.19 ^C^	33.77 ± 0.2 ^C^
Protein	6.69 ± 0.01 ^A^	8.32 ± 0.02 ^B^	8.97 ± 0.01 ^C^	11.82 ± 0.03 ^D^
Ash	1.36 ± 0.03 ^A^	1.6 ± 0.01 ^B^	1.7 ± 0 ^C^	2 ± 0 ^D^
**Mineral content (mg/Kg)**				
Ca	126.69 ± 2.37 ^A^	195.13 ± 6.1 ^B^	228.67 ± 0.63 ^BC^	251.52 ± 13.9 ^C^
Fe	5.12 ± 0.04 ^A^	14.87 ± 0.29 ^B^	20.67 ± 0.19 ^C^	23.75 ± 1.67 ^C^
K	1680.33 ± 24.83 ^A^	1760.52 ± 34.54 ^A^	2376.48 ± 21.82 ^B^	2678.76 ± 120.81 ^C^
Mg	168.54 ± 1.88 ^A^	239.69 ± 7.23 ^B^	284.01 ± 5.73 ^C^	315.27 ± 11.22 ^C^
Mn	3.28 ± 0.01 ^A^	5.74 ± 0.1 ^B^	6.94 ± 0.07 ^C^	7.76 ± 0.36 ^C^
P	634.29 ± 13.97 ^A^	1114.82 ± 31.23 ^B^	1303 ± 23.45 ^C^	1475.2 ± 58.15 ^D^
S	715.05 ± 12.98 ^A^	899.47 ± 33.23 ^B^	908.32 ± 14.21 ^B^	988.85 ± 35.21 ^B^
Zn	5.77 ± 0.23 ^A^	11.85 ± 0.35 ^B^	12.87 ± 0.12 ^BC^	14.91 ± 0.87 ^C^

**Table 4:** Calculated recipe’s digestibilities and PDCAASs of wheat and BVF-added breads. * The protein’s contribution to the recipe (%) was calculated as the grams of protein in the food ingredient divided by the total protein in the recipe and multiplied by 100 [50].

	Source of Protein	Wheat Bread	12% BVF Bread	25% BVF Bread	40% BVF Bread
Protein’s Contribution to the Recipe (%) *	BVF	0.00	15.32	31.15	41.99
	Gluten	94.81	80.29	65.28	55.00
	Yeast	5.19	4.39	3.57	3.01
Recipe’s Protein Digestibility		96.73	89.57	82.17	77.11
Recipe’s PDCAAS		24.55	28.45	32.48	35.24

**Table 5:** Canavanine contents of BV breads. Values are expressed as means ± standard errors of triplicate determination. Within columns, means with different letters are significantly different (*p* > 0.05). All the results are presented on a dry basis. * The canavanine contents in all the breads were calculated for the wet-basis results (42% moisture after baking).

Canavanine (mg/100 g)	12% BVF Bread	25% BVF Bread	40% BVF Bread
Flour Mix	10.75 ± 0.2 ^A^	22.03 ± 0.91 ^A^	35.52 ± 1.21 ^A^
Bread Crust	1.84 ± 0.01 ^C^	3.78 ± 0.25 ^C^	6.07 ± 0.64 ^C^
Bread Crumb	6.04 ± 0.71 ^B^	12.31 ± 0.92 ^B^	20.07 ± 1.13 ^B^
Bread *	3.87 ± 0.08 ^D^	7.89 ± 0.66 ^D^	12.86 ± 1.03 ^D^

## Data Availability

The original contributions presented in the study are included in the article, further inquiries can be directed to the corresponding author.
